# Supplementary material for: Quantum transports in two-dimensions with long range hopping
Source: Sci Rep. 2023 Apr 8;13:5763. doi: 10.1038/s41598-023-32888-8 (PMC10082852; doi:10.1038/s41598-023-32888-8)
Supplement: Supplementary file 1 — Supplementary Information. [file 41598_2023_32888_MOESM1_ESM.pdf]

Supplementary Material on Quantum Transports in Two-Dimensions with Long Range Hopping

Si-Si Wang[1,2,3], Kangkang Li[4], Yi-Ming Dai[1], Hui-Hui Wang[1,3], Yi-Cai Zhang[1], and Yan-Yang Zhang[1,3,2]

[1]School of Physics and Materials Science, Guangzhou University, 510006 Guangzhou, China
[2]School of Mathematics and Information Science, Guangzhou University, 510006 Guangzhou, China
[3]Huangpu Research and Graduate School of Guangzhou University, 510700 Guangzhou, China
[4]Department of Physics, Zhejiang Normal University, Jinhua 321004, China
[*]yanyang@gzhu.edu.cn

ABSTRACT

This supplementary material shows the scaling of conductance for the 1D long range model.

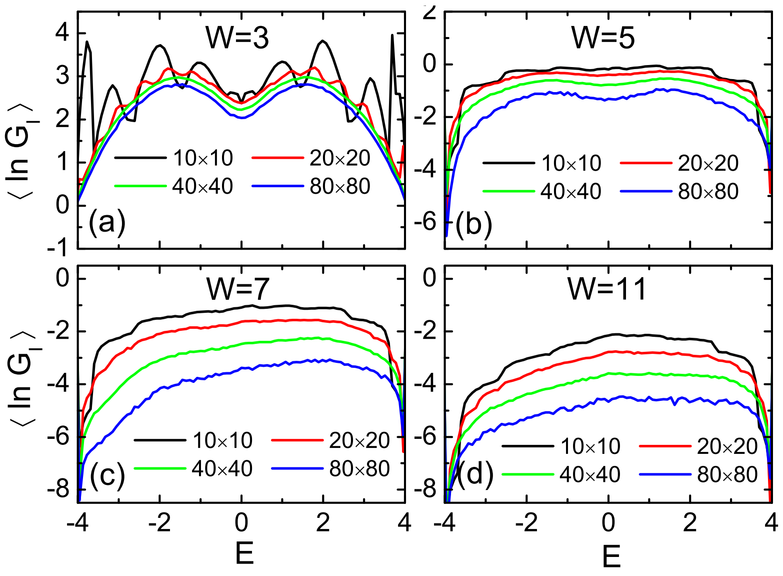

Figure S1. (Color online) Disorder averaged $\langle \ln G_\mathrm{I} \rangle$ as a function of Fermi energy E for different sample sizes: 10×10 (black), 20×20 (red), 40×40 (green), 80×80 (blue), at different disorder strengths: (a) $W = 3$, (b) $W = 5$, (c) $W = 7$, (d) $W = 11$. The average is over 1000 samples for the largest size and 5000 samples for other sizes.

In this Supplementary Material, we display the scaling results for weak disorder. In the extremely weak disorder regime ($W = 3$) shown in Figure S1 (a), there are significant finite-size fluctuations for the smallest size (10×10, black curve). After they are smoothed out in large sizes, it is clear that $\ln G_\mathrm{I}$ is decreasing with increasing sample size, in the whole energy region. This decreasing is more significant at larger disorder strengths, as shown in the rest panels of Figure S1, indicating a strong trend of localization. There seem to be some curve crossings around two ends of the energy interval. We have checked that this only occurs for the smallest size 10×10, and that for larger sizes, $\ln G_\mathrm{I}$ is still monotonically decreasing. Therefore, within the best numerical capability we can achieve, all band states with $W \geqslant 3$ is shown to be localized.
